# Metagenomic analysis demonstrates distinct changes in the gut microbiome of Kawasaki diseases children

**DOI:** 10.3389/fimmu.2024.1416185

**Published:** 2024-07-22

**Authors:** Linli Han, Xu Liu, Yue Lan, Yimin Hua, Zhenxin Fan, Yifei Li

**Affiliations:** ^1^ Department of Pediatrics, Ministry of Education Key Laboratory of Women and Children’s Diseases and Birth Defects, West China Second University Hospital, Sichuan University, Chengdu, Sichuan, China; ^2^ Key Laboratory of Bioresources and Ecoenvironment (Ministry of Education), College of Life Sciences, Sichuan University, Chengdu, Sichuan, China

**Keywords:** Kawasaki disease, metagenomic analysis, IVIG resistance, coronary artery lesions, diagnosis, prognosis

## Abstract

**Background:**

Kawasaki disease (KD) has been considered as the most common required pediatric cardiovascular diseases among the world. However, the molecular mechanisms of KD were not fully underlined, leading to a confused situation in disease management and providing precious prognosis prediction. The disorders of gut microbiome had been identified among several cardiovascular diseases and inflammation conditions. Therefore, it is urgent to elucidate the characteristics of gut microbiome in KD and demonstrate its potential role in regulating intravenous immunoglobulin (IVIG) resistance and coronary artery injuries.

**Methods:**

A total of 96 KD children and 62 controls were enrolled in the study. One hundred forty fecal samples had been harvested from KD patients, including individuals before or after IVIG treatment, with or without early coronary artery lesions and IVIG resistance. Fecal samples had been collected before and after IVIG administration and stored at −80°C. Then, metagenomic analysis had been done using Illumina NovaSeq 6000 platform. After that, the different strains and functional differences among comparisons were identified.

**Results:**

First, significant changes had been observed between KD and their controls. We found that the decrease of *Akkermansia muciniphila*, *Faecalibacterium prausnitzii*, *Bacteroides uniformis*, and *Bacteroides ovatus* and the increase of pathogenic bacteria *Finegoldia magna*, *Abiotrophia defectiva*, and *Anaerococcus prevotii* perhaps closely related to the incidence of KD. Then, metagenomic and responding functional analysis demonstrated that short-chain fatty acid pathways and related strains were associated with different outcomes of therapeutic efficacies. Among them, the reduction of *Bacteroides thetaiotaomicron*, the enrichment of *Enterococcus faecalis* and antibiotic resistance genes had been found to be involved in IVIG resistance of KD. Moreover, our data also revealed several potential pathogenetic microbiome of that KD patients with coronary artery lesions

**Conclusion:**

These results strongly proved that distinct changes in the gut microbiome of KD and the dysfunction of gut microbiomes should be responsible for the pathogenesis of KD and significantly impact the prognosis of KD.

## Introduction

Kawasaki disease (KD) has been considered as the most common required pediatric cardiovascular diseases among the world, with unknown illness cause (1). The lesions of coronary arteries, including coronary artery dilation, thrombosis, and aneurysms, which were secondary to KD, contributed to healthy conditions along all childhood, leading to high morbidities (2). Once it developed into a giant coronary aneurysm, it was highly associated with cardiac sudden death. Due to the limited understanding of the etiology of KD, the incidence of coronary artery injuries still remained as a level of 3%–5% among all KD patients, although several versions of guidelines have been issued to provide integrative management strategy for KD. Generally, acute and continuous inflammation attack was considered as the major pathophysiological changes in KD. So that, to underline the mechanisms of such inflammation attacks would benefit in attenuating the systematic injuries and reduce the risks of cardiac involvements. Due to severe inflammation attacks, multiple organ damages could be observed in part of KD cases, including gastrointestinal, kidney, lung, and nervous system injuries (3). According to previous researches, the incidence of coronary artery aneurysms could be reduced from 25% to approximately 4% by treating timely with intravenous immunoglobulin (IVIG) (4). However, the unexpected IVIG resistance prohibited its protective effectiveness to coronary artery (5, 6). At the same time, the elevated incidence of incomplete (atypical) KD also increased the difficulty in initially diagnosing KD, which had been supposed to be associated with enhanced inflammation attacks (7). Therefore, it was urgent to demonstrate the molecular mechanisms in inducing KD onsets. Moreover, the different regulating mechanisms between patients with or without coronary artery lesions, as well as IVIG resistance, were required to be addressed.

Recently, a series of studies presented that the dysregulation of gut microbes was related to many diseases, including cardiovascular diseases (8), diabetes (9), inflammatory bowel diseases (10), allergies (11), and cancers (12). It was identified that the impaired proportion of gut microbiota would induce metabolic hemostasis changing with unwilling metabolites accumulation, triggering inflammation activities. Thus, the dysregulation of gut microbiome had been proved to be tightly associated with autoimmune diseases and metabolic disorders. Moreover, some small sample size cohort studies of KD patients attempted to establish the relationship between gut microbiome and KD pathogenesis. A metagenomic analysis of 28 KD patients reported that the Ruminococcus bacteria and five Streptococcus species were recorded increasing during the acute and non-acute phase of KD, respectively (13). Another study based on 16S amplicon sequencing of five KD patients found that the *Fusobacteria*, *Shigella*, and *Streptococcus* were suppressed after immunoglobulin therapy (14). In animal studies, the use of probiotic *Clostridium butyricum* would significantly increase the abundance of bacteria, which produced short chain fatty acids (SCFAs) in KD mouse models and alleviated coronary artery injuries and reduced inflammatory response (15).

Although the above researches provided an initial correlation of gut microbiome disorders with KD onset. However, all the published studies failed to demonstrate the evidences that gut microbiota should be responsible for coronary artery involvement and IVIG resistance in human cohorts. Moreover, the microbiome sequencing methods used in previous researches seem to remain as a lower convinced test effectiveness with a very limited sample size, lacked a satisfied clinical decision supporting. Herein, we enrolled connectively 96 KD patients and 62 healthy controls for metagenomic analysis. Also, the stool samples had been collected before and after IVIG administration, which was able to explore the changes of gut microbiome associated with IVIG. Moreover, the relative larger sample cohort provided enough cases to reveal the differences of gut microbiome in KD patients with and without coronary lesions. So that, this research carried a comprehensive analysis of KD via metagenomic analysis, which provided more essential understanding of gut microbiome in participating the molecular mechanisms in KD pathogenesis and prognosis.

## Materials and methods

### Study design

The study was approved by the Ethics Committee of West China Second University Hospital of Sichuan University (No. 2020–092). The participants involved in this research had been enrolled from the West China Second University Hospital of Sichuan University from August 2022 to June 2023. A total of 96 consecutive KD children (53 males/43 females, average aged 3.1 ± 2.3 years) and 62 volunteer controls (28 males/34 females, average aged 4.2 ± 2.9 years) had been included for stool samples collection, and 140 in total (**Supplementary Table 1**). The enrolled controls were age-appropriate and sex-matched healthy children who were totally absent from history of KD.

### Inclusion and exclusion criteria

We used the following inclusion criteria to recruit candidates for further analysis: (1) all the patients should meet the diagnostic criteria for complete or incomplete diagnostic standards recommended by AHA (2017) for diagnosis, treatment, and long-term management of KD, and the diagnosis should be confirmed by two physicians; (2) coronary artery aneurysms were identified during acute or subacute phase by echocardiography; (3) programmed questionnaires, basic essential information, clinical manifestation, results of hematological examinations, therapeutic procedure, and echocardiography results were well collected; (4) the age of included patients varied from 1 year to 10 years, which is the most popular age for KD onset, to easy balance the bias from high-risk ages; and (5) the characteristics of coronary arteries were evaluated neither by transthoracic echocardiography or transcatheter angiography. The exclusion criteria included the following: (1) patients demonstrated any cardiovascular malformation; (2) patients had been diagnosed with an autoimmune disease before KD onset; (3) patients had received anticoagulant or antiplatelet medication before KD onset; (4) patients underwent any cardiac surgery; (5) myocarditis had been suspected before KD; (6) glucocorticoids had been provided before IVIG; (7) monoclonal antibody, including tumor necrosis factor (TNF)-α or interleukin (IL)-6 antibodies, was provided; (8) macrophage activation syndrome or hemophagocytic lymphohistiocytosis was diagnosed due to KD; (9) no available echocardiographic record within acute and subacute phases of KD.

### Therapeutic procedure and coronary artery assessment

All patients with KD were treated with high-dose IVIG (2 g/kg given as a single intravenous infusion) combined with 30–50 mg/kg/day high-dose aspirin. Those with recrudescent or persistent fever for ≥36h after the end of the first dose of IVIG infusion were treated with a second dose of 2 g/kg IVIG. Methylprednisolone (30 mg/kg/day for 3 consecutive days) followed by oral prednisone tapered over 7 days would be considered after the second IVIG administration. IVIG resistance was defined as persistent or recurrent fever (temperature of ≥38.0°C orally) or other clinical signs of KD for at least 36h but not >7 days after the initial IVIG. The patients were discharged from the hospital after their temperature remained normal for >48h and hematological examination returned to normal values. All echocardiographs were performed by two well-trained pediatric physicians. The physicians involved in the examination of enrolled patients were blinded to the clinical manifestation of receivers. The first echocardiography was performed before IVIG administration. The second echocardiography was performed during the subacute phase or before hospital discharge. The dilations of coronary arteries were calculated by *Z*-score >2, according to AHA KD management guideline. Either first or second echocardiography assessments identified dilation of coronary artery would be treated as the patient with coronary artery injury.

### Samples collection and metagenomic analysis

#### Sample collection

The fecal samples had been collected rapidly at the first day once a convinced diagnosis of KD reached, while following fecal samples would be collected at the 36th hour after initial IVIG administration. Thus, the fecal samples could be harvested before and after IVIG therapy. Before collection, the scope and methods were fully informed to patients’ parents by researchers. The children’s feces were collected only after their parents formally signed an informed consent to be participated in the study. Moreover, the parents could choose to provide feces either before or after IVIG administration, or both time points. All fecal samples were collected via sterile fecal sampler or disposable swab. The whole process was sterile and fast. After sampling, fecal samples were collected and frozen at −80°C for next step.

#### DNA extraction, assembly, functional prediction, and quantifcation of genes

First, the steps of DNA extraction and sequencing were as follows: the swabs or fecal sampler of total DNA was extracted using a Tiangen DNA Stool Mini Kit (Tiangen Biotech (Beijing) Co., Ltd., China) and sent to Novogene (Beijing, China) for sequencing using the Illumina NovaSeq 6000 platform with a paired-end sequencing length of 150 bp. Second, in order to remove the adapters and low-quality raw reads, the Trimmomatic based on a four-base-wide sliding window was used by average quality per base >20 and minimum length 90 bp after sequencing (16). Then, to eliminate host contamination, Bowtie2 (17), as part of the KneadData pipeline (https://github.com/biobakery/kneaddata), was used to remove the people potential sequences with the human reference genome (assembly GRCh38). *De-novo* assembly of each metagenomic samples from the quality-filtered Illumina reads was separately performed using MEGAHIT (18) with the option “-t 96 –m 0.95 –min-contig-len 300.” After assembling, gene prediction was performed using Prodigal (19) with the option “-p meta –g 11.” Using CD-HIT (20) and setting the “-c 0.95-aS 0.90” option, a non-redundant gene set with a threshold of 95% similarity and 90% query sequence coverage was constructed. Quantification of the non-redundant genes in each metagenome was performed using Salmon (21) with the option “—meta.” Total abundance of all genes, which mapped to the same gene type, was determined to the total abundance of each gene type. The non-redundant genes were further translated into amino acid sequences, using DIAMOND (22) to select “–id 80% –query-cover 70% –evalue 1e-5” in the Carbohydrate-Active enZYmes (CAZy) database (23).

#### Metagenomic analysis

To further explore differences in species and function, the abundances of gene family and microbial metabolic pathway were assessed by using HUMANn3 (24) with the ChocoPhlAn and UniRef90 EC filtered databases (25), respectively, and were normalized by copies per million (CPMs). And then, the taxonomic labels of metagenomic sequences were assigned using Kraken2 (26) with the option “–use-mpa-style.” Taxon abundances were normalized by relative abundance. Species markers were identified by using linear discriminant analysis effect size (LEfSe) (27) (LDA >2). To understand species diversity and variability between groups in species level, the alpha (α) diversity and beta (β) diversity were, respectively, calculated by the QIIME2 diversity plugin (28) and the QIIME2 plugin DEICODE (29). Furthermore, the time series analysis was drawn by using Mfuzz R Package (30) to reflect how changes among different groups. And the antibiotic resistance genes (ARGs) were quantified using ShortBRED (31). Differences in taxon, metabolic pathway abundances and ARGs were determined using LEfSe.

## Results

### Clinical parameters and sequencing data

A total of 96 KD children (53 males/43 females) and 62 controls (28 males/34 females) were enrolled in the study. One hundred forty fecal samples had been harvested from 95 KD patients before and after IVIG administration. Among them, 62 pieces of samples were collected from individuals before IVIG treatment (Bef-IVIG group), while 78 pieces of samples were collected after initial therapy (Aft-IVIG group). In order to further explore the relationship between the incidence of coronary artery injury before IVIG administration and intestinal microorganisms during acute KD, we retrieved the echocardiographic assessments of 62 KD patients with fecal samples collected before initial therapy. Therefore, there were 22 patients with early coronary artery lesions (CAL group), and 40 patients absent from such injuries (non-CAL group). In addition, among the 78 pieces of samples which were collected after IVIG administration, 21 samples from IVIG-resistant patients (IVIG-R group), and 57 samples from IVIG-sensitive patients (IVIG-S). According to the comparisons setup, the species annotation and functional annotation were performed in the fecal samples between healthy control group (Con), the KD disease group before and after IVIG treatment (Bef-IVIG/Aft-IVIG), the group with or without coronary artery injury (CAL/non-CAL), and the group with or without IVIG resistance (IVIG-R/IVIG-S), in order to identify the pathogenic microbiotic strains, which could induce the onset and prognosis of KD.

### Differences in gut microbiota composition and function between the KD patients before and after treatment

**Figure 1:**
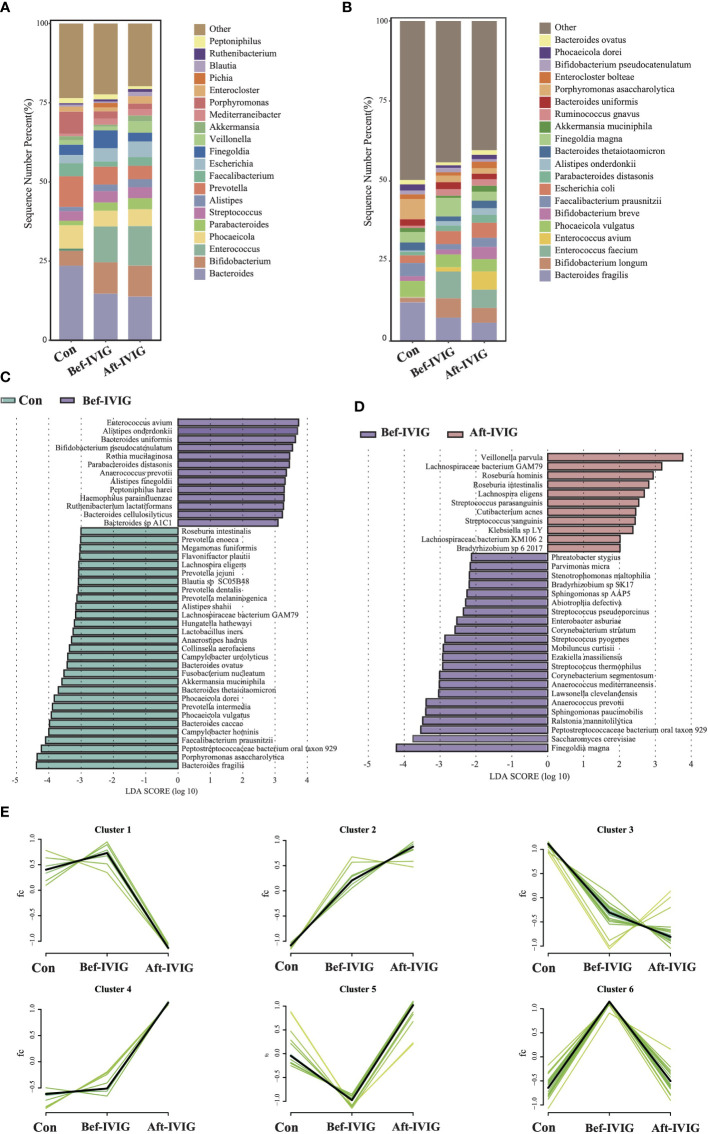
Differences in gut microbiota composition between the KD patients before and after treatment. **(A)** The gut microbial composition in three groups at genus level. **(B)** The gut microbial composition in three groups at species level. **(C)** LEfSe analysis between control and Bef-IVIG groups. **(D)** LEfSe analysis between Bef-IVIG and Aft-IVIG groups. **(E)** Time series analysis in three groups.

In order to identify the crucial strains participating in the pathogenesis of KD disease and impacting its treatment, we systematically analyzed three sets of metagenomic data based on the time for fecal samples collection, as group control (healthy group), Bef-IVIG (KD patients before treatment) and Aft-IVIG (KD patients after treatment). The α-diversity (Shannon and Simpson indexes) in the Bef-IVIG and Aft-IVIG groups were significantly lower than that in the control group (**Supplementary Figures S1A**, **S1B**, *p* < 0.001), while there was no significant change between Bef-IVIG and Aft-IVIG groups (*p* > 0.05). We also performed Principal Coordinate Analysis (PCoA), and there was no significant difference between the three groups (**Supplementary Figure S1C**, *p* > 0.05). Compared to the control group, the dominant phyla in the Bef-IVIG group changed from Bacteroidetes (48.53%), Firmicutes (31.08%), and Proteobacteria (10.95%) to Firmicutes (36.06%), Bacteroidetes (32.41%), and Actinobacteria (13.91%). Moreover, the relative abundances of Firmicutes were increased further after IVIG treatment (**Supplementary Figure S1D**). At genus level, the relative abundances of *Bacteroides* in the Bef-IVIG and Aft-IVIG groups showed a large decrease compare to the control group, while the relative abundances of *Bifidobacterium* and *Enterococcus* in the Bef-IVIG and Aft-IVIG groups showed a large increase (**Figure 1A**). Notably, compare to the control group, the relative abundances of *Finegoldia* increased in the Bef-IVIG group and returned to the normal level compared to control group in the Aft-IVIG group. Moreover, *Finegoldia magna* as a member of *Finegoldia*, which was reported able to induce inflammation by activating neutrophils, showed a similar change (32). Moreover, the relative abundances of *Akkermansia muciniphila (* 33), *Faecalibacterium prausnitzii (* 34), *Bacteroides thetaiotaomicron (* 35), and *Bacteroides ovatus (* 36), which reported as probiotics, were decreased in the Bef-IVIG group and returned to the control group level in the Aft-IVIG group (**Figure 1B**). Next, we performed differential analysis to demonstrate the differential microbes by using LEfSe. Consistent with the above results, *Akkermansia muciniphila*, *Faecalibacterium prausnitzii*, *Bacteroides thetaiotaomicron*, and *Bacteroides ovatus* were recorded significantly decreased in the Bef-IVIG group (**Figure 1C**, *p* < 0.05). In addition, *Enterococcus avium* was significantly increased in the Bef-IVIG group and showed the highest LDA score (*p* < 0.05). Comparing to the Bef-IVIG group, *Finegoldia magna* was significantly decreased in the Aft-IVIG group and showed the highest LDA score (**Figure 1D**, *p* < 0.05).

To further demonstrate the change trend of differential microbes among control group, Bef-IVIG group and Aft-IVIG group, we performed time series analysis by using Mfuzz. As shown in **Figure 1E**, a total of six clusters were obtained. Among them, cluster 5 showed a reduction firstly from control to Bef-IVIG and then elevation from Bef-IVIG to Aft-IVIG, including *Akkermansia muciniphila*, *Faecalibacterium prausnitzii*, *Bacteroides uniformis*, and *Bacteroides ovatus*. On the contrary, cluster 6 showed an opposite trend of increase firstly from control to Bef-IVIG and then decrease from Bef-IVIG to Aft-IVIG, including *Finegoldia magna*, *Abiotrophia defective*, and *Anaerococcus prevotii.* These results meant that the increase of these pathogenic strains and the decrease of these beneficial bacteria in KD patients, while the decrease of pathogenic bacteria and the recovery of beneficial bacteria after treatment which like control group.

**Figure 2:**
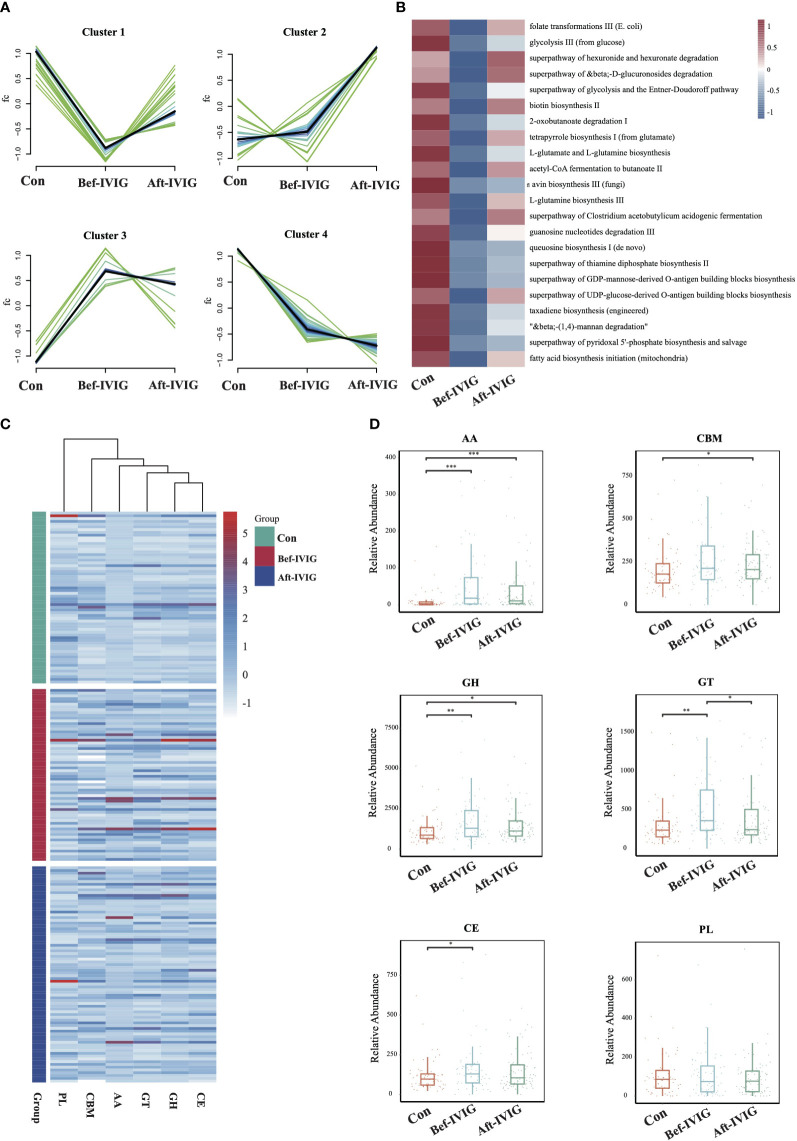
Differences in gut microbiota function between the KD patients before and after treatment. **(A)** Time series analysis of KEGG pathways. **(B)** The differential KEGG pathways of cluster1. **(C)** The distribution of CAZy enzymes in each sample. **(D)** Differential analysis of gut microbial CAZy enzymes. CBMs, carbohydrate-binding module; GTs, glycosyl transferases; PLs, polysaccharide lyases; AA, auxiliary activity enzymes; GH, glycoside hydrolases; CE, carbohydrate esterases. *, **, *** means P< 0.05, P< 0.01, P< 0.005, respectively.

To compare the functional differences between the three groups, we performed functional annotation and differential analysis. A total of 87 pathways with significant differences were identified between control and Bef-IVIG groups, and 26 pathways with significant differences were identified between Bef-IVIG and Aft-IVIG groups (**Supplementary Figures S2A**, **S2B**). The results of time series analysis for all differential pathways showed four main trends, and 22 differential pathways were included in cluster 1, which showed a trend of decrease first and then increase from control to Bef-IVIG to Aft-IVIG, such as acetyl-CoA fermentation to butanoate II, superpathway of Clostridium acetobutylicum acidogenic fermentation, folate transformations III (E. coli), and biotin biosynthesis II (**Figure 2A**). And the heat map showed similar results (**Figure 2B**). Next, we investigated the alteration of carbohydrate-active enzymes (CAZy) between the three groups (**Figure 2C**). Compared to the control group, the abundance of carbohydrate esterases (CEs), glycosyl transferases (GTs), auxiliary activity enzymes (AA), and glycoside hydrolases (GH) families were significantly up-regulated in the Bef-IVIG group (*p* < 0.05). However, compared to the Bef-IVIG group, the abundance of AA, CBM, CE, GH, and GT all decreased in the Aft-IVIG group which same as control group, but only GT was significantly different from the Bef-IVIG group (**Figure 2D**, *p* < 0.05). These results showed that the carbohydrate metabolism pathway of the gut microbiota was more active in KD patients. In addition, the analysis result of ARGs showed that the abundance of AAC (6’)-Ii, *efrA*, eatAv, PC1 beta-lactamase (*blaZ*), and EC-19 were significantly upregulated in the Bef-IVIG group (**Supplementary Figure S2C**, *p* < 0.05). Compared to the Bef-IVIG group, the abundance of *tetA*(46), *dfrF*, and *mdeA* were significantly up-regulated after treatment (**Supplementary Figure S2D**, *p* < 0.05). But the number of ARGs was reduced in KD patients after treatment, indicating that the treatment had a certain effect.

### Differences in gut microbiota composition and function between the KD patients with or without coronary artery injury

**Figure 3:**
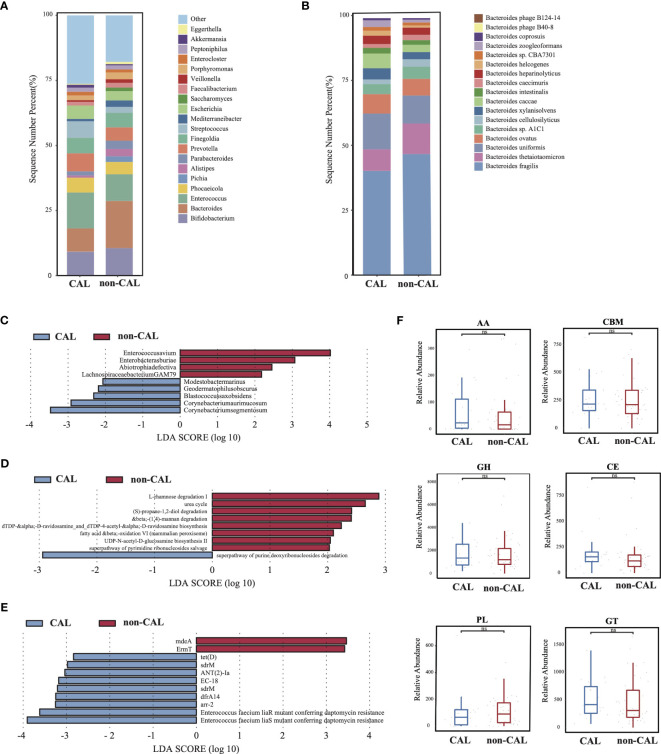
Differences in gut microbiota composition and function between the KD patients with or without coronary artery injury. **(A)** The gut microbial composition between CAL and non-CAL groups at genus level. **(B)** The gut microbial composition between CAL and non-CAL groups at species level. **(C)** LEfSe analysis of microbiota between CAL and non-CAL groups. **(D)** LEfSe analysis of KEGG pathways between CAL and non-CAL groups. **(E)** LEfSe analysis of ARGs between CAL and non-CAL groups. **(F)** Differential analysis of gut microbial CAZy enzymes between CAL and non-CAL groups. ns, not signficance.

As shown in **Supplementary Figures S1EG**, there was no significantly difference in microbial diversity and structure between the CAL (KD patients with coronary artery injury) and non-CAL groups (KD patients without coronary artery injury) (*p* > 0.05). The dominant phyla in the CAL group were Firmicutes (40.84%), Bacteroidetes (26.01%), and Actinobacteria (16.67%), while the dominant phyla in the non-CAL group were Bacteroidetes (37.21%), Firmicutes (34.14%), and Proteobacteria (12.05%) (**Supplementary Figure S1H**). At genus level, compare to the non-CAL group, *Bacteroides* showed a large decrease in the CAL group, and this difference was contributed by *Bacteroides thetaiotaomicron* which was a probiotic (**Figures 3A, B**). Furthermore, nine microbes were identified as differential microbes, including *Enterococcus avium*, *Enterobacter asburiae*, *Abiotrophia defective*, and *Lachnospiraceae bacterium* GAM79 were significantly increased in the non-CAL group (**Figure 3C**, LDA>2, *p* < 0.05). And the abundance of *Corynebacterium segmentosum*, *Corynebacterium aurimucosum*, *Blastococcus saxobsidens*, *Modestobacter marinus*, and *Geodermatophilus obscurus* were significantly decreased in the non-CAL group (*p* < 0.05). We obtained nine differential pathways by the differential analysis between the two groups, and only superpathway of purine deoxyribonucleosides degradation was enriched in the CAL group (**Figure 3D**, *p* < 0.05). Compare to the non-CAL group, more ARGs up-regulated in the CAL group, including *tet(D*), *sdrM*, *ANT(2)-Ia*, *EC-18*, *sdrM*, *dfrA14*, *arr-2*, *Enterococcus faecium liaR* mutant conferring daptomycin resistance, and *Enterococcus faecium liaS* mutant conferring daptomycin resistance (**Figure 3E**, *p* < 0.05), which were different from control, Bef-IVIG and Aft-IVIG group. In addition, there was no significantly difference in all CAZy families between the two groups (**Figure 3F**, *p* > 0.05).

### The patients of IVIG resistance have different gut microbiota composition and function compared with non-IVIG resistance

**Figure 4:**
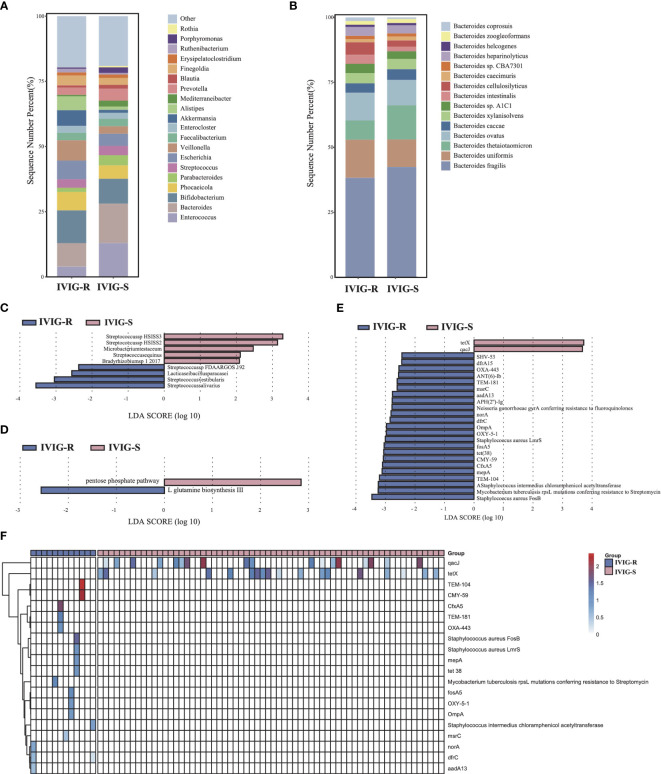
Differences in gut microbiota composition and function between the KD patients with or without IVIG resistance. **(A)** The gut microbial composition between IVIG-R and IVIG-S groups at genus level. **(B)** The gut microbial composition between IVIG-R and IVIG-S groups at species level. **(C)** LEfSe analysis of microbiota between IVIG-R and IVIG-S groups. **(D)** LEfSe analysis of KEGG pathways between IVIG-R and IVIG-S groups. **(E)** LEfSe analysis of ARGs between IVIG-R and IVIG-S groups. **(F)** The distribution of differential ARGs in each sample.

There was no significantly difference in microbial diversity and structure between the IVIG resistance (IVIG-R) and non-IVIG resistance groups (IVIG-S) (**Supplementary Figures S1I–K**, *p* > 0.05). The dominant phyla in both IVIG-R and IVIG-S groups were Firmicutes, Bacteroidetes, and Actinobacteria (**Supplementary Figure S1L**). Compared with IVIG-S groups, there were many differences at genus level, especially, the abundance of *Bacteroides* and *Enterococcus* were decreased significantly in the IVIG-R group, and this difference was contributed by *Bacteroides thetaiotaomicron* and *Enterococcus faecalis*, respectively (**Figures 4A, B**), indicating that the accumulation of pathogenic bacteria *Enterococcus faecalis* and the reduct of *Bacteroides thetaiotaomicron* in KD maybe led to the resistance of IVIG. The LEfSe results showed that *Streptococcus salivarius*, *Streptococcus vestibularis*, *Lacticaseibacillus paracasei*, and *Streptococcus* sp. FDAARGOS 192 were significantly increased in the IVIG-R group (**Figure 4C**, *p* < 0.05), which were all associated with endocarditis. This result showed that these pathogenic strains were also likely important factors leading to resistance to IVIG in KD patients. By KEGG analysis, only two pathways were identified as differential pathways between the two groups, including pentose phosphate pathway, which was riched in IVIG-S group and L-glutamine biosynthesis III which was riched in IVIG-R group (**Figure 4D**, *p* < 0.05). In total of 25 ARGs were significantly changed between the two groups, and 23 ARGs were significantly upregulated in the IVIG-R group (**Figure 4E**, *p* < 0.05), suggesting that the increase of ARGs was another reason of IVIG resistance in KD patients. Notably, we found that *qacJ* and *tetX* were distributed in most samples of the IVIG-S group, but none of the samples in the IVIG-R group contained the two ARGs (**Figure 4F**). There was no significantly difference in all CAZy families between the two groups (**Supplementary Figure S2E**, *p* > 0.05). These finds implied that the enrichment of pathogenic strains and ARGs in KD patients induced the complexity of treatment and ultimately aggravates the condition.

## Discussion

KD, an acute febrile systemic vasculitis in children, had already become the most common cause of acquired heart disease (4). Recently, the change of the gut microbiota had been observed among KD patients (13, 14, 37). However, poor study design and limited sample size failed to address its characteristics and its role in KD pathogenesis. In this work, we found that the dysregulation of gut strains and the changes of metabolic pathway were highly associated with KD through metagenomic analysis.

Several strains had been observed to be altered significantly during process of KD. *Finegoldia magna*, one Gram-positive anaerobic which colonized the skin and other non-sterile body surfaces, was closely related with acute necrotizing pancreatitis (38), endocarditis (39), infected foot ulcers (40), necrotising fasciitis (41), spinal intramedullary abscesses (42), and so on. As an important opportunistic pathogen, *F. magna* was thought to be some virulence factors to acts as an infectious agent. For example, the protein L, which presented the high affinity to bind immunoglobulin light chains, and SufA (subtilisin-like protease), which could degrade host defense proteins LL-37 and MIG/CXCL9. In addition, FAF (*F. magna* adhesion factor) mediated adhesion by binding to galectin 7 (a keratinocyte marker). Also, one report showed that *F. magna* strains ALB8 (coding protein FAF), 312 (coding protein L) and 505 (naturally lacking proteins for FAF and L) as well as their associated proteins all could active the neutrophils to induce the inflammation, which dependent on acting rearrangement, NADPH oxidases and the ERK1/2 pathway, showing the *F. magna* serving as an activator of neutrophils and involving in inflammatory responses. *Enterococcus faecalis* is a species of Enterococci bacteria typically found in the gastrointestinal tract, oral cavity, and vaginal tract. While *E. faecalis* is generally considered non-pathogenic in healthy individuals, it can opportunistically cause urinary tract infections, wound infections, bacteremia, endocarditis, and intra-abdominal and pelvic infections in immunocompromised individuals. *Abiotrophia defectiva* is a commensal strain present in the human oral cavity, intestine, and urinary tract (43). However, under conditions of compromised immunity, it may contribute to infectious endocarditis, often associated with serious complications such as spondylodiscitis and brain abscess (44). Additionally, *Anaerococcus prevotii* has been linked to septic arthritis and brain abscesses, while *Streptococcus vestibularis* has been implicated in schizophrenia and chronic obstructive pulmonary disease (45–49). Most of the pathogenic strains had been suspected to be involved in inflammation responses and severe infections. Thus, the identified strains might participate in the dysregulation of immune activities and inflammation attacks on KD onset.

In contrast to pathogenic strains, probiotics play a protective role in various diseases. *Akkermansia muciniphila*, emerging as a promising probiotic candidate, is renowned for its potential benefits in inflammatory bowel disease (33), obesity, diabetes, atherosclerosis, and lethal sepsis (50). This strain, known for its mucin-degrading capabilities, densely populates the intestinal epithelium. Studies involving 4-week administration of *A. muciniphila* in mice have indicated its role in promoting the proliferation of Lgr5+ intestinal stem cells (ISCs) and enhancing the differentiation of Paneth cells and goblet cells in the small intestine and colon, thereby contributing to intestinal homeostasis maintenance (51). Mechanistic research on *A. muciniphila* has revealed its involvement in immune regulation, maintenance of intestinal mucosal barrier function, regulation of intestinal dysbiosis, and inhibition of colonization by other pathogens in inflammatory bowel disease (33). Additionally, phospholipids from *A. muciniphila’s* cell membrane have been shown to preferentially induce the expression of certain inflammatory cytokines, thus modulating host immune homeostasis (52). Recent studies have introduced a novel tripeptide RKH derived from live *A. muciniphila*, which demonstrates the potential to alleviate sepsis-induced mortality and organ damage. RKH peptide exerts its effects by reducing inflammatory cell activation and the overproduction of proinflammatory factors, and by binding to Toll-like receptor 4 (TLR4) to modulate TLR4 signal transduction in immune cells (50).

*Faecalibacterium prausnitzii* is recognized as one of the pivotal bacteria in the human gut microbiota, notable for its production of butyric acid (34) and its anti-inflammatory properties (53). Increasing evidence suggests that the abundance of F. prausnitzii is upregulated in various conditions including hyperlipidemia, prediabetes, type II diabetes, non-alcoholic fatty liver disease, and inflammatory bowel disease (54–57). Consequently, *F. prausnitzii* has garnered significant attention as a promising next-generation probiotic. In the realm of antitumor applications, *F. prausnitzii* has demonstrated efficacy in ameliorating immune checkpoint inhibitor (ICI)–induced colitis, restoring gut microbial composition, and enhancing the antitumor activity of immunotherapy in murine models (58). Efforts to harness the potential of *F. prausnitzii* have led to the development of mixed cultures, isolated from the feces of healthy individuals. These mixed cultures have shown to increase the survival rate of *F. prausnitzii* and enhance butyrate production. Furthermore, strategies to mitigate *F. prausnitzii’s* sensitivity to oxygen have been explored, with oxygen tolerance experiments demonstrating efficacy. These findings have been corroborated in both animal and human studies, further supporting the potential therapeutic applications of *F. prausnitzii* (36). *Bacteroides uniformis*, a prominent member of the genus Bacteroides, plays a crucial role in both the gut and vaginal microbiomes. Recognized as a butyrate-producing bacterium, *B. uniformis* is generally associated with maintaining health (59, 60). A strain of B. uniformis (CECT 7771), isolated from the gut microbiota of infants, has undergone safety testing as a probiotic in mice (60) and is currently under consideration for clinical studies in humans (61). Notably, *B. uniformis* has been inversely correlated with serum LDL-C levels, which are implicated in the acceleration of atherosclerosis (62, 63). Additionally, specific strains of *B. uniformis*, such as PF-BaE8 and PF-BaE13, in collaboration with *B. coprocola* AS101, have demonstrated comprehensive anti-inflammatory properties and improvement in intestinal barrier function (64). *Bacteroides ovatus*, also recognized as a new generation probiotic (65), has been extensively investigated for its role in intestinal immunity (66), tumor treatment (67), and dietary fiber metabolism (68). Decreased abundances of *B. ovatus* in the gut have been observed in patients with coronary artery disease (CAD) complicated by nonalcoholic fatty liver disease (NAFLD) compared to CAD patients without NAFLD (69). *Bacteroides thetaiotaomicron*, one of the most abundant bacteria in the gut microbiome, colonizes the gel-like mucus layer of intestinal epithelial cells (IECs) and aids in the degradation of complex polysaccharides and the maturation of the host immune system (35, 70). *Streptococcus salivarius*, a symbiotic microorganism found in the digestive tract, skin, breast milk, and body fluids of humans, exhibits biosafety and a close relationship with *Streptococcus thermophilus (* 71). Consequently, *S. salivarius* holds promise as a potential oral probiotic candidate (72). Thus, the reduction of probiotics would enhance the damages secondary to inflammation attacks from KD, which was considered to be tightly associated with KD onset and coronary artery injuries.

At the species level, the abundance of opportunistic pathogens *Finegoldia magna*, *Abiotrophia defectiva*, *Anaerococcus prevotii*, along with the probiotics *Akkermansia muciniphila*, *Faecalibacterium prausnitzii*, *Bacteroides uniformis*, and *Bacteroides ovatus*, exhibited differential regulation compared to the control group before IVIG treatment (**Figures 1B, D**). Remarkably, following IVIG treatment, the abundance of these strains reverted to levels comparable to those of healthy controls (**Figure 1E**, **Supplementary Table 1**). These findings suggest that the dysregulation of upregulated pathogens, which contribute to inflammation, and downregulated probiotics, which modulate inflammation, may be pivotal in the pathogenesis and treatment of KD. Moreover, the results underscore the complex interplay among these strains. Further analysis revealed a significant reduction in the abundance of *Bacteroides thetaiotaomicron* in KD patients with coronary artery injury compared to those without, independent of IVIG treatment. This finding suggests a potential role for *B. thetaiotaomicron* in coronary artery injury unrelated to IVIG treatment. Similarly, decreased abundance of *B. thetaiotaomicron* was associated with IVIG resistance in KD patients, coinciding with a significant increase in the pathogenic strain *Enterococcus faecalis*. These results highlight the potential significance of *B. thetaiotaomicron* in influencing therapeutic strategies and treatment outcomes in KD. Additionally, the presence of *Enterococcus faecalis*, *Streptococcus salivarius*, *Streptococcus vestibularis*, and *Lacticaseibacillus paracasei* appears to complicate the management of KD.

Biotin, also known as vitamin H or B7, serves as an essential cofactor for biotin-dependent enzymes involved in various biological processes, including gluconeogenesis, fatty acid synthesis, and amino acid metabolism (73). Deficiency in the intestinal stem cell (ISC)–specific biotin transporter, Smvt, has been shown to impair intestinal maintenance and ISC mitosis (74). Suboptimal circulating biotin levels have been observed in severe obesity, along with altered biotin-related gene expression in human adipose tissue, suggesting a link between severe obesity, bacterial biotin production, and host metabolism (75). However, biotin biosynthesis was found to be decreased in KD before IVIG treatment, indicating potential limitations in these biological processes in children with KD. Butyrate-producing bacteria play a critical role in maintaining gut homeostasis and epithelial integrity, with butyrate itself contributing to cardiovascular health by reducing blood pressure, improving ischemia-reperfusion injury, and decreasing the risk of CAD and atherosclerosis (76). Notably, the acetyl-coenzyme A pathway, a key pathway for butyrate synthesis, was downregulated in KD, suggesting a potential reduction in butyrate levels as a contributing factor in the development of the disease (77). Purine nucleotide degradation, which produces uric acid, has been associated with various syndromes, including immune deficiency, myopathy, kidney stones, hyperuricemia, and gout. The superpathway of purine deoxyribonucleosides degradation was enriched in KD patients with coronary artery injury, consistent with clinical manifestations such as anemia and hypoxia following coronary artery injury (78). L-glutamine (Gln) serves as a substrate for DNA, ATP, protein, and lipid synthesis, playing a crucial role in cardiovascular physiology and pathology by exerting antioxidant and anti-inflammatory effects (79). Supplementation with Gln has been shown to prevent cardiometabolic diseases such as pulmonary arterial hypertension (80), ischemia-reperfusion injury (81), and heart failure (82). In KD patients with IVIG resistance, upregulation of L-glutamine biosynthesis III suggests activation of a protective mechanism to mitigate heart damage through antioxidant and anti-inflammatory effects.

The carbohydrate-active enzymes database, which covers the data on their genetic, structural, mechanistic, and functional information is divided into six classes, named the glycoside hydrolases(GH), the glycosyl transferases (GT), the polysaccharide lyases (PL), the carbohydrate esterases (CE), the auxiliary active (AA), and the carbohydrate-binding modules (CBM) (23). And all the six classes had been measured among all involved analyses, and significant changes could be observed along with IVIG treatment. GTs as one of carbohydrate-active enzymes catalyze the activation of sugars *in vivo* to bind to different receptor molecules, such as proteins, nucleic acids, oligosaccharides, lipids, and small molecules. The products of glycosylation have many biological functions. Moreover, protein O-GlcNAcylation has been found to be multiply involved in the progression of cardiovascular dysfunction which depends on the unique disease environment (83), including the heart failure, cardiac hypertrophy (84), diabetic heart disease (85), ischemia reperfusion injury (86), and pulmonary artery hypertension (87). In addition, other families of CAZh L-glutamine (Gln) serve as a substrate for DNA, ATP, protein, and lipid synthesis, playing a crucial role in cardiovascular physiology and pathology by exerting antioxidant and anti-inflammatory effects. Supplementation with Gln has been shown to prevent cardiometabolic diseases such as pulmonary arterial hypertension, ischemia-reperfusion injury, and heart failure (88, 89). In KD patients with IVIG resistance, upregulation of L-glutamine biosynthesis III suggests activation of a protective mechanism to mitigate heart damage through antioxidant and anti-inflammatory effects.

ARGs encode proteins or other molecular components present in the genomes of bacteria or other microorganisms, conferring resistance to antibiotics. Our analysis revealed significant upregulation of 23 ARGs in KD patients with IVIG resistance, underscoring the role of antibiotic resistance in the development of IVIG resistance. Among these ARGs, QacJ belongs to the multidrug-resistant transporter family, predominantly found in Staphylococcus species (90). Numerous reports have demonstrated the efficacy of immunoglobulin and its subclasses in treating Staphylococcus infections (91). Thus, even in KD patients without IVIG resistance, the enrichment of the qacJ resistance gene may not hinder the alleviation of KD symptoms with IVIG treatment. The *tetX* gene, which encodes a FAD- and NADPH-requiring oxidoreductase, confers resistance to tetracycline in strict anaerobic bacteria such as Bacteroides. *Bacteroides fragilis*, a member of the Bacteroides genus, relies on IgA responses to establish stable colonization in the gut, excluding exogenous competitors from occupying mucosal niches. Consequently, we observed enrichment of Bacteroides expressing *tetX* in the IVIG-resistant KD group. While both q*acJ* and *tetX* were present in the KD patients without IVIG resistance, strains harboring *qacJ* were susceptible to inhibition by IVIG treatment, whereas strains containing *tetX* were more prone to colonization despite IVIG treatment (92).

## Conclusion

In our study, we observed a decrease in probiotic species such as *Akkermansia muciniphila*, *Faecalibacterium prausnitzii*, *Bacteroides uniformis*, and *Bacteroides ovatus*, alongside an increase in pathogenic bacteria including *Finegoldia magna*, *Abiotrophia defectiva*, and *Anaerococcus prevotii*, which may be closely associated with KD incidence. Treatment was shown to reverse the abundance of these strains, with *Finegoldia magna* appearing to be particularly influential. Additionally, compared to the untreated group, the short-chain fatty acid pathways associated with gut microbes were upregulated post-treatment, as revealed by KEGG analysis. Furthermore, we observed a reduction in *Bacteroides thetaiotaomicron* and an enrichment of *Enterococcus faecalis* and ARGs in KD patients, likely contributing to IVIG resistance. *Bacteroides thetaiotaomicron*, a probiotic reduced in KD, was found to play a crucial role in KD-induced coronary complications. Similarly, other pathogenic bacteria such as *Enterococcus avium*, *Enterobacter asburiae*, *Abiotrophia defectiva*, and *Lachnospiraceae bacterium* GAM79 appeared to have similar implications. These findings suggest that different gut strains may influence KD incidence, treatment responsiveness, and complication rates, highlighting a significant correlation between gut microbes and KD. Future experiments will be conducted to validate these findings.

## Data availability statement

The original contributions presented in the study are publicly available. This data can be found here: PRJNA1112983 (SRA).

## Ethics statement

The study was approved by the Ethics Committee of West China Second University Hospital of Sichuan University (NO. 2020-092). The studies were conducted in accordance with the local legislation and institutional requirements. Written informed consent for participation in this study was provided by the participants’ legal guardians/next of kin.

## Author contributions

LH: Conceptualization, Data curation, Formal analysis, Investigation, Methodology, Resources, Software, Validation, Writing – original draft. XL: Formal analysis, Investigation, Methodology, Resources, Software, Validation, Visualization, Writing – original draft. YL: Investigation, Methodology, Software, Writing – original draft. YH: Conceptualization, Project administration, Supervision, Writing – review & editing. ZF: Conceptualization, Methodology, Project administration, Software, Supervision, Writing – review & editing. YFL: Conceptualization, Formal analysis, Funding acquisition, Investigation, Methodology, Project administration, Supervision, Writing – review & editing.

## Supplementary material

Supplementary Figure 1
**(A)** Alpha diversity estimates (Shannon index) in three groups. **(B)** Alpha diversity estimates (Simpson index) in three groups. **(C)** Principal Coordinate Analysis (PCoA) in three groups. **(D)** The gut microbial composition in three groups at phylum level. **(E)** Alpha diversity estimates (Shannon index) between CAL and non-CAL groups. **(F)** Alpha diversity estimates (Simpson index) between CAL and non-CAL groups. **(G)** Principal Coordinate Analysis (PCoA) between CAL and non-CAL groups. **(H)** The gut microbial composition between CAL and non-CAL groups at phylum level. **(I)** Alpha diversity estimates (Shannon index) between IVIG-R and IVIG-S groups. **(J)** Alpha diversity estimates (Simpson index) between IVIG-R and IVIG-S groups. **(K)** Principal Coordinate Analysis (PCoA) between IVIG-R and IVIG-S groups. **(L)** The gut microbial composition between IVIG-R and IVIG-S groups at phylum level.

Supplementary Figure 2
**(A)** LEfSe analysis of KEGG pathways between control and Bef-IVIG groups. **(B)** LEfSe analysis of KEGG pathways between Bef-IVIG and Aft-IVIG groups. **(C)** LEfSe analysis of ARGs between control and Bef-IVIG groups. **(D)** LEfSe analysis of ARGs between Bef-IVIG and Aft-IVIG groups. **(E)** Differential analysis of gut microbial CAZy enzymes between IVIG-R and IVIG-S groups.

Supplementary Table 1 List of differential microbes among control, Bef-IVIG and Aft-IVIG group for six groups of time series analysis.
