# Supplementary figures and images for: Metagenomic analysis demonstrates distinct changes in the gut microbiome of Kawasaki diseases children
Source: Front Immunol. 2024 Jul 22;15:1416185. doi: 10.3389/fimmu.2024.1416185 (PMC11298399; doi:10.3389/fimmu.2024.1416185)

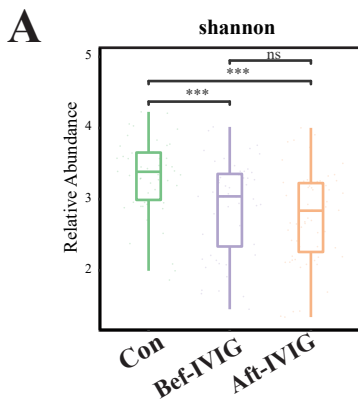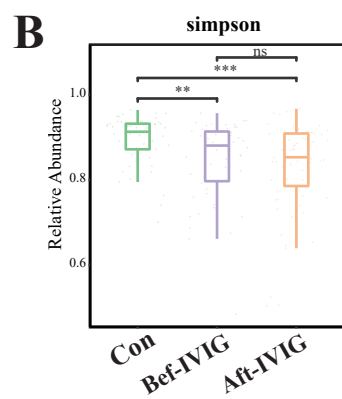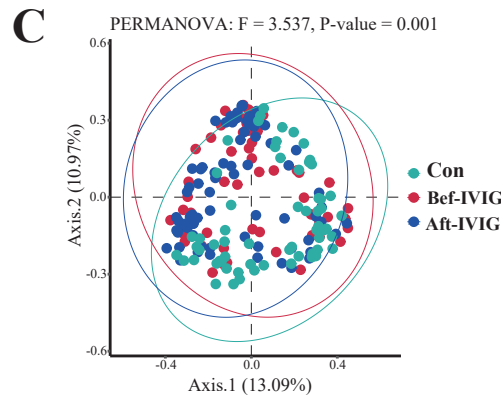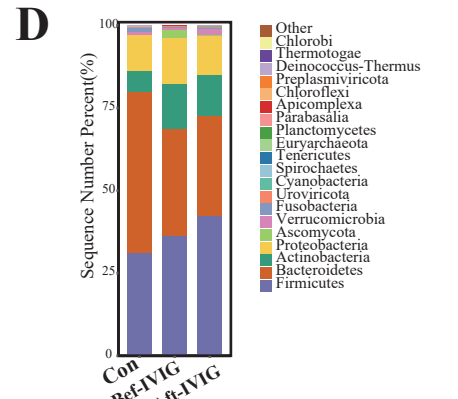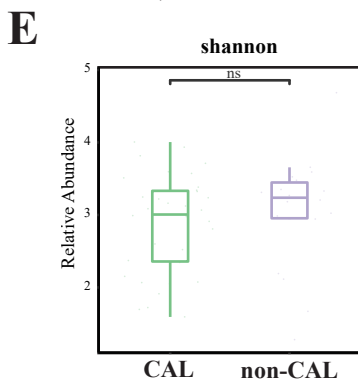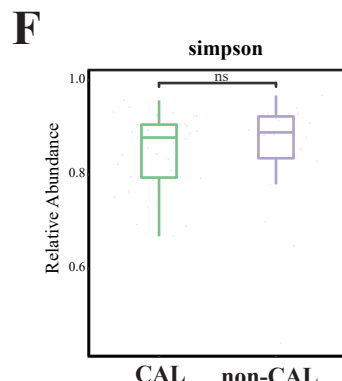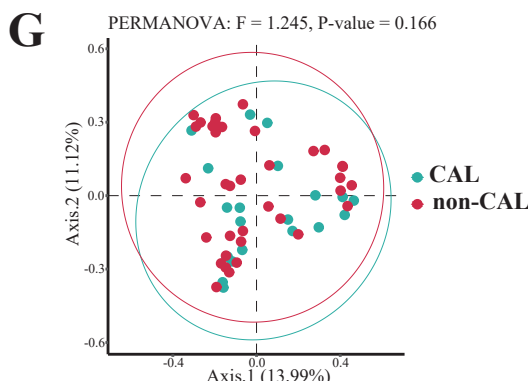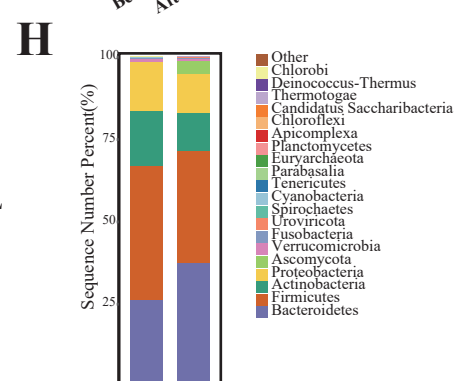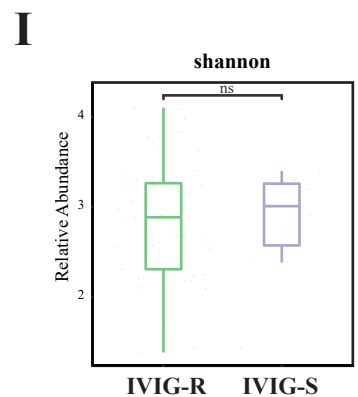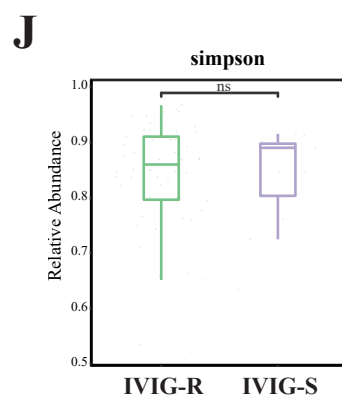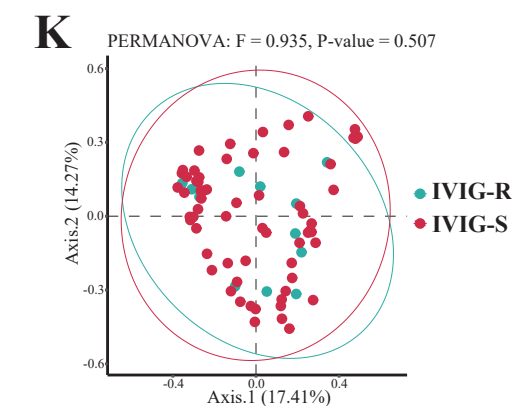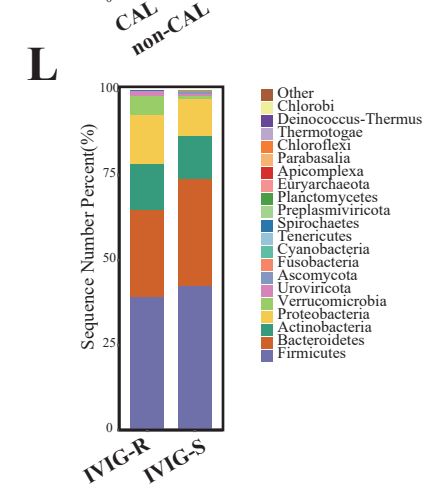

Supplement: Supplementary Figure 1 — (A) Alpha diversity estimates (Shannon index) in three groups. (B) Alpha diversity estimates (Simpson index) in three groups. (C) Principal Coordinate Analysis (PCoA) in three groups. (D) The gut microbial composition in three groups at phylum level. (E) Alpha diversity estimates (Shannon index) between CAL and non-CAL groups. (F) Alpha diversity estimates (Simpson index) between CAL and non-CAL groups. (G) Principal Coordinate Analysis (PCoA) between CAL and non-CAL groups. (H) The gut microbial composition between CAL and non-CAL groups at phylum level. (I) Alpha diversity estimates (Shannon index) between IVIG-R and IVIG-S groups. (J) Alpha diversity estimates (Simpson index) between IVIG-R and IVIG-S groups. (K) Principal Coordinate Analysis (PCoA) between IVIG-R and IVIG-S groups. (L) The gut microbial composition between IVIG-R and IVIG-S groups at phylum level. [file Image_1.pdf]

A

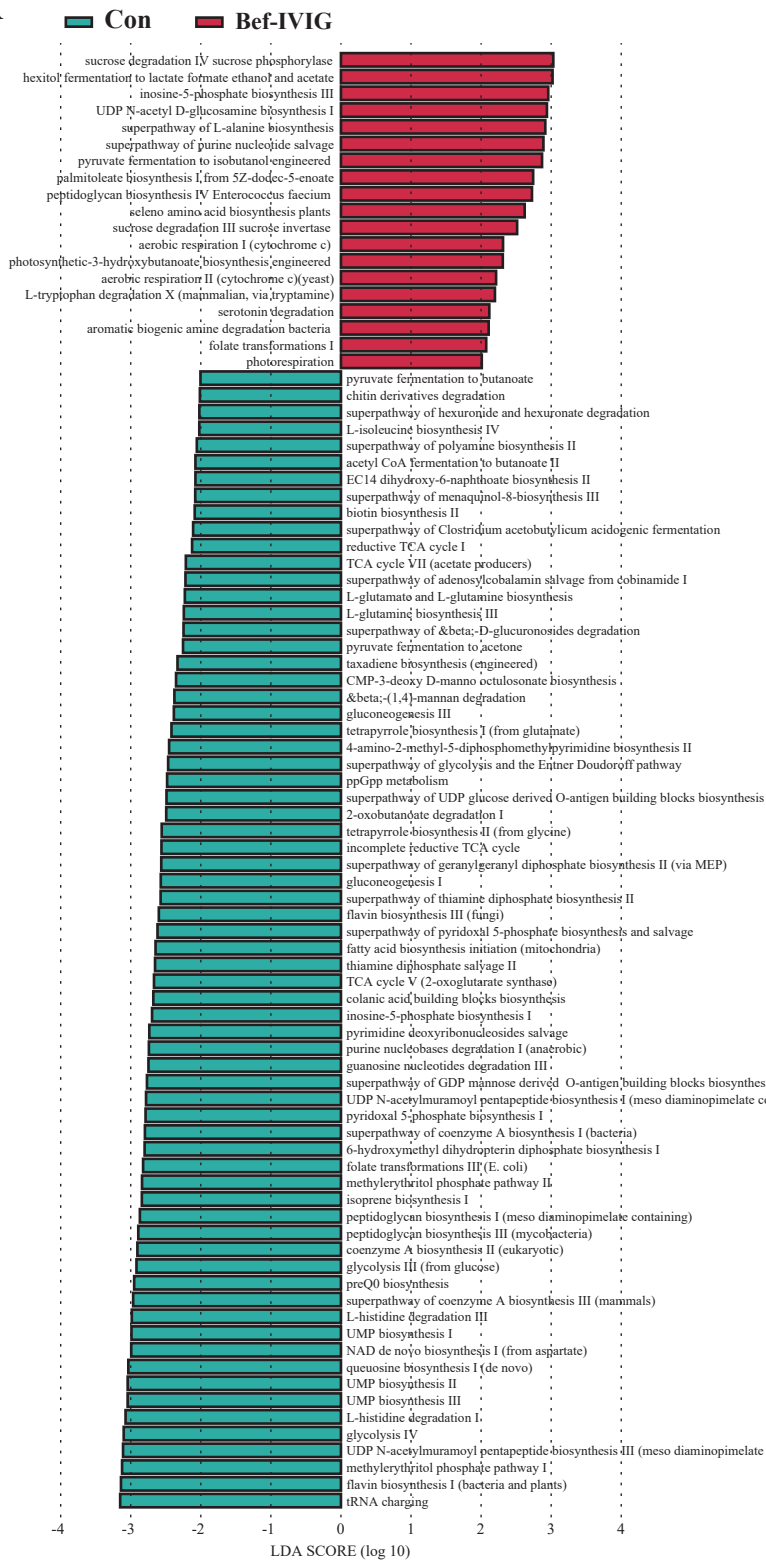

B

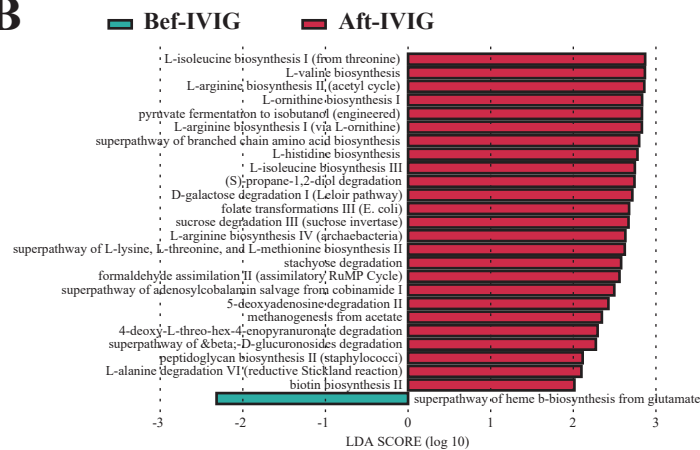

C

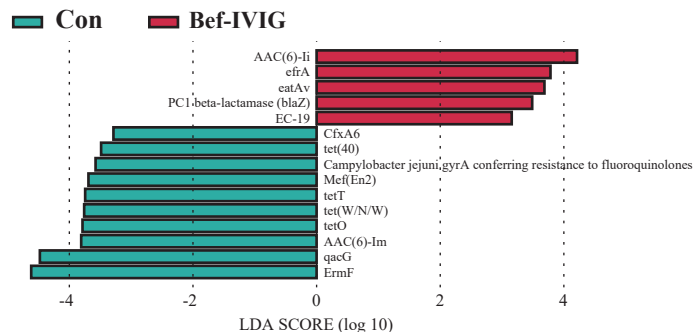

D

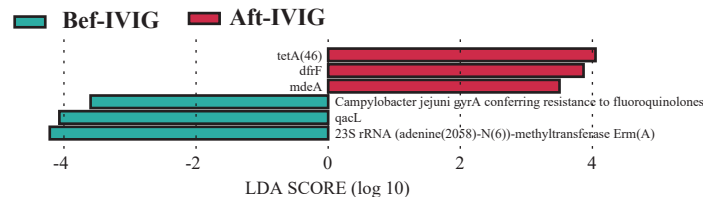

E

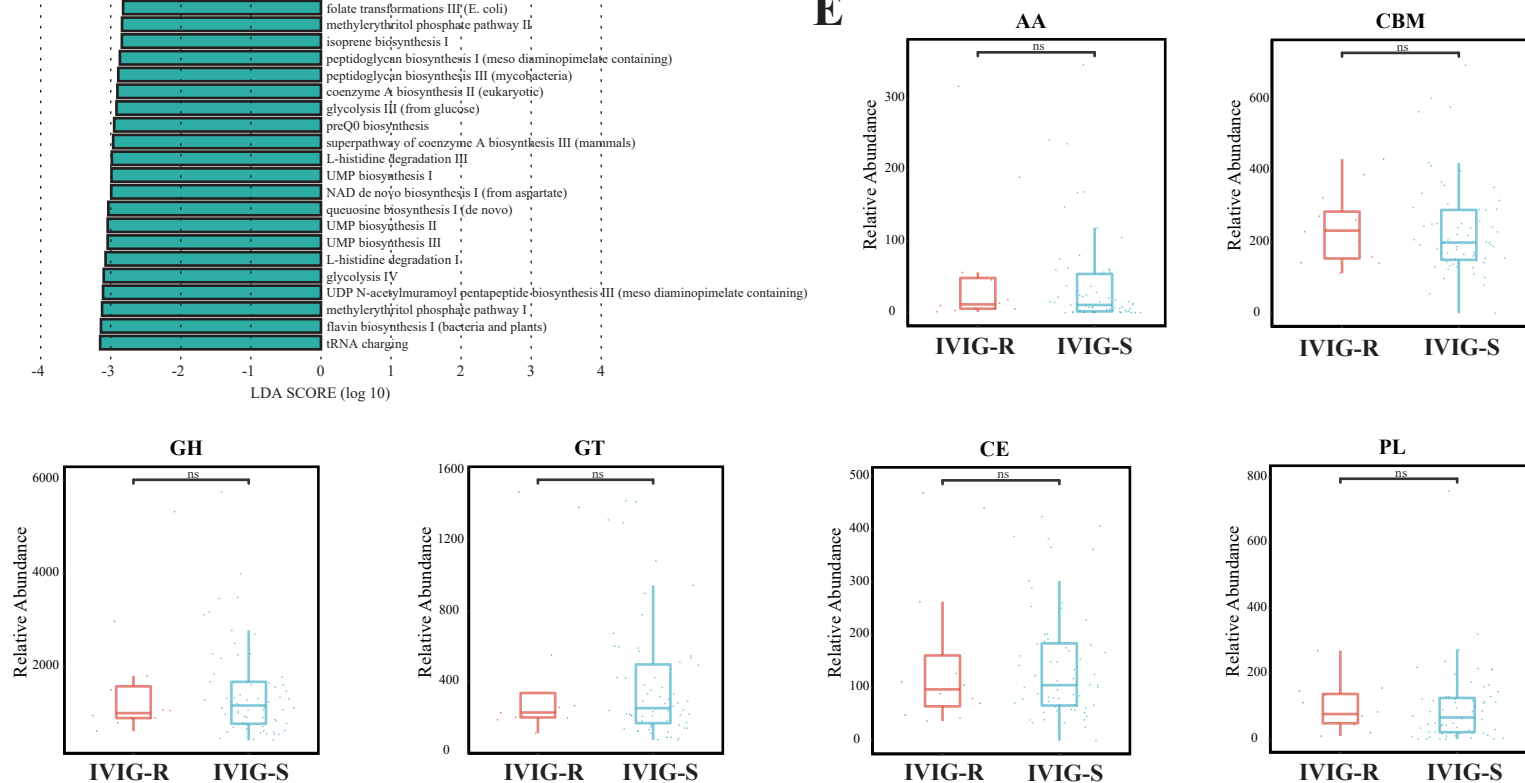

Supplement: Supplementary Figure 2 — (A) LEfSe analysis of KEGG pathways between control and Bef-IVIG groups. (B) LEfSe analysis of KEGG pathways between Bef-IVIG and Aft-IVIG groups. (C) LEfSe analysis of ARGs between control and Bef-IVIG groups. (D) LEfSe analysis of ARGs between Bef-IVIG and Aft-IVIG groups. (E) Differential analysis of gut microbial CAZy enzymes between IVIG-R and IVIG-S groups. [file Image_2.pdf]
