# Supplementary material for: Metagenomic analysis demonstrates distinct changes in the gut microbiome of Kawasaki diseases children
Source: Front Immunol. 2024 Jul 22;15:1416185. doi: 10.3389/fimmu.2024.1416185 (PMC11298399; doi:10.3389/fimmu.2024.1416185)
Supplement: Supplementary file 4 [file Table_1.docx]

**Supplementary Table 1.** Detail information for each studied sample from involved participants in the research.

| Number | Category | Sample | Age  (year) | Sex | Weight  (kg) | Hight  (cm) | Z scores | Once or Second IVIG infusion |
| --- | --- | --- | --- | --- | --- | --- | --- | --- |
| 1 | KD | **KD001** | 2.0 | female | 8.5 | 85 | <2 | once |
| 2 | KD | **KD002** | 2.0 | female | 8.5 | 85 | <2 | once |
| 3 | KD | **KD003** | 0.7 | female | 7.5 | 66 | 1 | once |
| 4 | KD | **KD004** | 0.7 | female | 7.5 | 66 | 1 | once |
| 5 | KD | **KD005** | 0.9 | female | 8.3 | 71 | 1 | once |
| 6 | KD | **KD006** | 0.9 | female | 8.3 | 71 | 1 | once |
| 7 | KD | **KD007** | 4.2 | male | 17 | 105 | <2 | once |
| 8 | KD | **KD008** | 4.4 | female | 17 | 100 | <2 | once |
| 9 | KD | **KD010** | 1.4 | female | 9.5 | 80 | <2 | once |
| 10 | KD | **KD011** | 1.4 | female | 9.5 | 80 | <2 | once |
| 11 | KD | **KD012** | 1.7 | female | 12.5 | 87 | <2 | once |
| 12 | KD | **KD013** | 1.7 | female | 12.5 | 87 | <2 | once |
| 13 | KD | **KD014** | 3.8 | male | 16 | 102 | <2 | once |
| 14 | KD | **KD015** | 2.5 | male | 13.5 | 95 | <2 | once |
| 15 | KD | **KD016** | 2.5 | male | 13.5 | 95 | 1 | second |
| 16 | KD | **KD017** | 1.0 | male | 11.5 | 79 | <2 | once |
| 17 | KD | **KD018** | 1.0 | male | 11.5 | 79 | <2 | second |
| 18 | KD | **KD020** | 5.6 | female | 25 | 124 | <2 | once |
| 19 | KD | **KD021** | 5.6 | female | 25 | 124 | <2 | once |
| 20 | KD | **KD022** | 5.1 | male | 19 | 112 | 1 | once |
| 21 | KD | **KD023** | 5.1 | male | 19 | 112 | 1 | once |
| 22 | KD | **KD025** | 4.6 | female | 20 | 120 | <2 | second |
| 23 | KD | **KD026** | 5.1 | male | 20 | 114 | <2 | once |
| 24 | KD | **KD027** | 9.9 | female | 26 | 133 | 1 | second |
| 25 | KD | **KD028** | 2.2 | female | 12 | 85 | 1 | once |
| 26 | KD | **KD029** | 2.2 | female | 12 | 85 | 1 | second |
| 27 | KD | **KD030** | 1.0 | male | 9.5 | 76 | 1 | once |
| 28 | KD | **KD032** | 3.9 | female | 14 | 98 | 1 | once |
| 29 | KD | **KD033** | 1.4 | male | 13 | 84 | 1 | once |
| 30 | KD | **KD034** | 6.2 | male | 22 | 116 | 1 | once |
| 31 | KD | **KD036** | 0.3 | female | 7.1 | 64 | 1 | once |
| 32 | KD | **KD037** | 0.5 | male | 8.3 | 67 | 1 | once |
| 33 | KD | **KD038** | 1.3 | male | 11 | 77 | <2 | second |
| 34 | KD | **KD041** | 5.2 | male | 16.8 | 106 | <2 | once |
| 35 | KD | **KD042** | 1.4 | male | 13 | 84 | 1 | once |
| 36 | KD | **KD043** | 0.3 | male | 7.5 | 64 | 1 | once |
| 37 | KD | **KD044** | 0.3 | male | 7.5 | 64 | 1 | once |
| 38 | KD | **KD045** | 3.7 | female | 16 | 104 | <2 | once |
| 39 | KD | **KD046** | 3.7 | female | 16 | 104 | <2 | second |
| 40 | KD | **KD047** | 1.3 | female | 11 | 80 | <2 | once |
| 41 | KD | **KD048** | 1.3 | female | 11 | 80 | <2 | once |
| 42 | KD | **KD049** | 3.3 | female | 15 | 97 | <2 | once |
| 43 | KD | **KD050** | 3.3 | female | 15 | 97 | <2 | once |
| 44 | KD | **KD051** | 3.4 | male | 16 | 104 | <2 | once |
| 45 | KD | **KD052** | 3.4 | male | 16 | 104 | <2 | once |
| 46 | KD | **KD053** | 3.4 | female | 14.5 | 98 | <2 | once |
| 47 | KD | **KD054** | 3.4 | female | 14.5 | 98 | <2 | once |
| 48 | KD | **KD055** | 2.3 | female | 13 | 91 | <2 | once |
| 49 | KD | **KD056** | 2.3 | female | 13 | 91 | <2 | once |
| 50 | KD | **KD057** | 0.5 | female | 8.1 | 69 | <2 | once |
| 51 | KD | **KD058** | 0.5 | female | 8.1 | 69 | <2 | once |
| 52 | KD | **KD059** | 2.5 | male | 14 | 92 | <2 | once |
| 53 | KD | **KD060** | 2.5 | male | 14 | 92 | <2 | once |
| 54 | KD | **KD061** | 0.7 | male | 10.5 | 67 | <2 | once |
| 55 | KD | **KD062** | 0.7 | male | 10.5 | 67 | <2 | once |
| 56 | KD | **KD063** | 8.1 | male | 26.5 | 135 | <2 | second |
| 57 | KD | **KD064** | 8.1 | male | 26.5 | 135 | <2 | second |
| 58 | KD | **KD065** | 3.3 | female | 17 | 102 | <2 | once |
| 59 | KD | **KD066** | 3.3 | female | 17 | 102 | <2 | once |
| 60 | KD | **KD067** | 0.9 | female | 8.5 | 69 | <2 | once |
| 61 | KD | **KD068** | 0.9 | female | 8.5 | 69 | <2 | once |
| 62 | KD | **KD069** | 2.8 | female | 14.7 | 97 | <2 | once |
| 63 | KD | **KD070** | 2.8 | female | 14.7 | 97 | <2 | once |
| 64 | KD | **KD071** | 5.3 | male | 26 | 120 | <2 | once |
| 65 | KD | **KD072** | 5.3 | male | 26 | 120 | <2 | once |
| 66 | KD | **KD073** | 1.1 | female | 14 | 74 | <2 | once |
| 67 | KD | **KD074** | 1.1 | female | 14 | 74 | <2 | once |
| 68 | KD | **KD075** | 9.5 | male | 27 | 137 | <2 | second |
| 69 | KD | **KD076** | 9.5 | male | 27 | 137 | <2 | second |
| 70 | KD | **KD077** | 1.6 | male | 11 | 83 | <2 | once |
| 71 | KD | **KD078** | 1.6 | male | 11 | 83 | <2 | once |
| 72 | KD | **KD079** | 1.2 | male | 9.5 | 75 | <2 | once |
| 73 | KD | **KD080** | 1.2 | male | 9.5 | 75 | <2 | once |
| 74 | KD | **KD081** | 1.3 | male | 13 | 82 | <2 | once |
| 75 | KD | **KD082** | 1.3 | male | 13 | 82 | <2 | once |
| 76 | KD | **KD083** | 1.4 | male | 11 | 67 | <2 | once |
| 77 | KD | **KD084** | 1.4 | male | 11 | 67 | <2 | once |
| 78 | KD | **KD085** | 3.9 | male | 17 | 110 | <2 | once |
| 79 | KD | **KD086** | 3.9 | male | 17 | 110 | <2 | once |
| 80 | KD | **KD087** | 8.4 | female | 26 | 129 | 1 | once |
| 81 | KD | **KD088** | 8.4 | female | 26 | 129 | 1 | once |
| 82 | KD | **KD089** | 2.2 | male | 12.5 | 90 | 1 | once |
| 83 | KD | **KD090** | 2.2 | male | 12.5 | 90 | 1 | once |
| 84 | KD | **KD091** | 3.8 | male | 16 | 104 | <2 | once |
| 85 | KD | **KD092** | 3.8 | male | 16 | 104 | <2 | once |
| 86 | KD | **KD093** | 0.9 | female | 9 | 75 | <2 | once |
| 87 | KD | **KD094** | 0.9 | female | 9 | 75 | <2 | once |
| 88 | KD | **KD095** | 0.2 | male | 4.8 | 55 | <2 | once |
| 89 | KD | **KD096** | 0.2 | male | 4.8 | 55 | <2 | once |
| 90 | KD | **KD097** | 1.3 | female | 11 | 75 | <2 | once |
| 91 | KD | **KD098** | 1.3 | female | 11 | 75 | <2 | once |
| 92 | KD | **KD099** | 0.4 | female | 8 | 63 | 1 | once |
| 93 | KD | **KD100** | 0.4 | female | 8 | 63 | 1 | once |
| 94 | KD | **KD101** | 1.4 | female | 9 | 76 | 1 | once |
| 95 | KD | **KD102** | 1.4 | female | 9 | 76 | 1 | once |
| 96 | KD | **KD103** | 3.5 | female | 17.5 | 98 | <2 | once |
| 97 | KD | **KD104** | 3.5 | female | 17.5 | 98 | <2 | once |
| 98 | KD | **KD105** | 2.3 | male | 12 | 83 | 1 | second |
| 99 | KD | **KD106** | 1.6 | male | 11.5 | 82 | 1 | second |
| 100 | KD | **KD107** | 6.2 | male | 22.5 | 121 | 1 | once |
| 101 | KD | **KD108** | 2.1 | male | 26 | 126 | 1 | once |
| 102 | KD | **KD109** | 4.8 | female | 18 | 110 | <2 | once |
| 103 | KD | **KD110** | 3.7 | female | 15.5 | 101 | <2 | once |
| 104 | KD | **KD111** | 7.1 | female | 22.5 | 125 | <2 | once |
| 105 | KD | **KD112** | 7.3 | female | 18 | 120 | <2 | second |
| 106 | KD | **KD113** | 6.3 | female | 20.5 | 124 | <2 | second |
| 107 | KD | **KD114** | 7.0 | male | 30 | 136 | 1 | once |
| 108 | KD | **KD115** | 5.5 | female | 21 | 116 | <2 | once |
| 109 | KD | **KD116** | 1.1 | male | 9.5 | 77 | <2 | once |
| 110 | KD | **KD117** | 2.3 | male | 12.5 | 91 | <2 | once |
| 111 | KD | **KD118** | 6.3 | female | 21.5 | 120 | <2 | once |
| 112 | KD | **KD119** | 3.0 | female | 14 | 98 | <2 | once |
| 113 | KD | **KD120** | 2.8 | male | 12 | 92 | 1 | second |
| 114 | KD | **KD121** | 1.7 | male | 10.5 | 80 | 1 | once |
| 115 | KD | **KD122** | 4.2 | female | 15 | 104 | <2 | once |
| 116 | KD | **KD123** | 3.5 | male | 20 | 105 | <2 | once |
| 117 | KD | **KD124** | 2.3 | male | 16 | 90 | <2 | once |
| 118 | KD | **KD125** | 2.4 | male | 15 | 92 | <2 | once |
| 119 | KD | **KD126** | 0.4 | female | 6 | 61 | <2 | second |
| 120 | KD | **KD127** | 1.8 | male | 12 | 85 | <2 | once |
| 121 | KD | **KD128** | 3.3 | male | 15.5 | 102 | <2 | once |
| 122 | KD | **KD129** | 4.5 | female | 15 | 100 | <2 | once |
| 123 | KD | **KD130** | 2.3 | male | 13 | 90 | <2 | second |
| 124 | KD | **KD131** | 2.8 | male | 16 | 98 | <2 | once |
| 125 | KD | **KD132** | 3.3 | male | 18 | 100 | 1 | once |
| 126 | KD | **KD133** | 0.1 | female | 4.8 | 51 | <2 | once |
| 127 | KD | **KD134** | 4.0 | male | 17 | 109 | <2 | once |
| 128 | KD | **KD135** | 0.5 | male | 8 | 67 | <2 | once |
| 129 | KD | **KD136** | 5.2 | male | 17 | 110 | <2 | once |
| 130 | KD | **KD137** | 3.2 | female | 14 | 94 | <2 | second |
| 131 | KD | **KD138** | 5.9 | male | 20 | 115 | <2 | second |
| 132 | KD | **KD139** | 4.8 | female | 18 | 110 | <2 | once |
| 133 | KD | **KD140** | 2.1 | male | 26 | 126 | <2 | once |
| 134 | KD | **KD141** | 0.3 | male | 7.5 | 66 | <2 | once |
| 135 | KD | **KD149** | 3.4 | male | 16 | 101 | <2 | once |
| 136 | KD | **KD150** | 11.3 | male | 55 | 158 | 1 | once |
| 137 | KD | **KD152** | 0.9 | male | 10 | 76 | 1 | second |
| 138 | KD | **KD153** | 3.2 | female | 16 | 106 | 1 | once |
| 139 | KD | **KD154** | 0.6 | female | 7.2 | 70 | 1 | once |
| 140 | KD | **KD155** | 1.2 | male | 9.8 | 75 | 1 | once |
| 141 | CK | **CK001** | 6.3 | female | 22 | 117 | / | / |
| 142 | CK | **CK003** | 0.7 | female | 6.9 | 65 | / | / |
| 143 | CK | **CK004** | 10.1 | female | 34 | 153 | / | / |
| 144 | CK | **CK005** | 4.9 | male | 16 | 109 | / | / |
| 145 | CK | **CK006** | 2.1 | female | 15 | 90 | / | / |
| 146 | CK | **CK007** | 11.2 | female | 39 | 154 | / | / |
| 147 | CK | **CK008** | 6.8 | female | 19.5 | 120 | / | / |
| 148 | CK | **CK009** | 0.7 | female | 10 | 64 | / | / |
| 149 | CK | **CK012** | 0.9 | male | 11 | 78 | / | / |
| 150 | CK | **CK013** | 1.8 | female | 9.5 | 80 | / | / |
| 151 | CK | **CK014** | 9.2 | female | 26 | 130 | / | / |
| 152 | CK | **CK015** | 5.8 | male | 22.5 | 126 | / | / |
| 153 | CK | **CK016** | 7.5 | male | 33 | 139 | / | / |
| 154 | CK | **CK017** | 7.1 | female | 19 | 119 | / | / |
| 155 | CK | **CK018** | 11.2 | female | 41.5 | 145 | / | / |
| 156 | CK | **CK019** | 1.8 | male | 12 | 85 | / | / |
| 157 | CK | **CK021** | 2.3 | female | 13.5 | 87 | / | / |
| 158 | CK | **CK022** | 7.7 | male | 26 | 133 | / | / |
| 159 | CK | **CK023** | 5.8 | female | 17.5 | 109 | / | / |
| 160 | CK | **CK024** | 0.5 | female | 8 | 65 | / | / |
| 161 | CK | **CK025** | 4.8 | female | 17 | 105 | / | / |
| 162 | CK | **CK026** | 1.0 | female | 8.1 | 68.5 | / | / |
| 163 | CK | **CK027** | 2.0 | female | 11 | 89 | / | / |
| 164 | CK | **CK028** | 2.0 | female | 12 | 89 | / | / |
| 165 | CK | **CK029** | 7.7 | female | 19 | 116 | / | / |
| 166 | CK | **CK030** | 2.5 | female | 12.5 | 92 | / | / |
| 167 | CK | **CK031** | 2.4 | female | 13.5 | 93 | / | / |
| 168 | CK | **CK032** | 2.3 | female | 13.5 | 87 | / | / |
| 169 | CK | CK033 | 3.4 | female | 16 | 101 | / | / |
| 170 | CK | CK034 | 2.8 | female | 12 | 91 | / | / |
| 171 | CK | CK035 | 2.4 | female | 13 | 92 | / | / |
| 172 | CK | CK036 | 5.9 | female | 22 | 118 | / | / |
| 173 | CK | CK037 | 5.6 | female | 15 | 110 | / | / |
| 174 | CK | CK038 | 3.8 | female | 17 | 95 | / | / |
| 175 | CK | CK039 | 7.0 | female | 19 | 123 | / | / |
| 176 | CK | CK040 | 3.5 | female | 14 | 100 | / | / |
| 177 | CK | CK041 | 3.3 | female | 12.5 | 97 | / | / |
| 178 | CK | CK042 | 0.8 | female | 10 | 75 | / | / |
| 179 | CK | CK043 | 0.9 | female | 7.1 | 70 | / | / |
| 180 | CK | CK044 | 2.5 | female | 16 | 95 | / | / |
| 181 | CK | CK045 | 7.1 | male | 32.5 | 138 | / | / |
| 182 | CK | CK046 | 2.8 | male | 14 | 95 | / | / |
| 183 | CK | CK047 | 4.8 | male | 17.5 | 110 | / | / |
| 184 | CK | CK048 | 9.0 | male | 22 | 120 | / | / |
| 185 | CK | CK049 | 1.8 | male | 13 | 86 | / | / |
| 186 | CK | CK050 | 3.3 | male | 101 | 16 | / | / |
| 187 | CK | CK051 | 4.2 | male | 16 | 109 | / | / |
| 188 | CK | CK052 | 0.3 | male | 8 | 63 | / | / |
| 189 | CK | CK053 | 4.8 | male | 19 | 108 | / | / |
| 190 | CK | CK054 | 2.0 | male | 12 | 87 | / | / |
| 191 | CK | CK055 | 10.8 | male | 31 | 141 | / | / |
| 192 | CK | CK056 | 6.5 | male | 21.5 | 118 | / | / |
| 193 | CK | CK057 | 6.2 | male | 14 | 95 | / | / |
| 194 | CK | CK058 | 6.0 | male | 19 | 120 | / | / |
| 195 | CK | CK059 | 4.8 | male | 16 | 106 | / | / |
| 196 | CK | CK060 | 5.7 | male | 17 | 113 | / | / |
| 197 | CK | CK061 | 1.3 | male | 11 | 81 | / | / |
| 198 | CK | CK062 | 2.3 | male | 11.5 | 89 | / | / |
| 199 | CK | CK063 | 1.6 | male | 11.5 | 84 | / | / |
| 200 | CK | CK064 | 2.6 | male | 12.5 | 94 | / | / |
| 201 | CK | CK065 | 4.8 | male | 19.5 | 118 | / | / |
| 202 | CK | CK066 | 0.5 | male | 9.2 | 77 | / | / |
